# Construction of Customized Sub-Databases from NCBI-nr Database for Rapid Annotation of Huge Metagenomic Datasets Using a Combined BLAST and MEGAN Approach

**DOI:** 10.1371/journal.pone.0059831

**Published:** 2013-04-01

**Authors:** Ke Yu, Tong Zhang

**Affiliations:** Environmental Biotechnology Laboratory, Department of Civil Engineering, The University of Hong Kong, Hong Kong SAR, China; Children’s Medical Research Institute, Australia

## Abstract

We developed a fast method to construct local sub-databases from the NCBI-nr database for the quick similarity search and annotation of huge metagenomic datasets based on BLAST-MEGAN approach. A three-step sub-database annotation pipeline (SAP) was further proposed to conduct the annotation in a much more time-efficient way which required far less computational capacity than the direct NCBI-nr database BLAST-MEGAN approach. The 1^st^ BLAST of SAP was conducted using the original metagenomic dataset against the constructed sub-database for a quick screening of candidate target sequences. Then, the candidate target sequences identified in the 1^st^ BLAST were subjected to the 2^nd^ BLAST against the whole NCBI-nr database. The BLAST results were finally annotated using MEGAN to filter out those mistakenly selected sequences in the 1^st^ BLAST to guarantee the accuracy of the results. Based on the tests conducted in this study, SAP achieved a speedup of ∼150–385 times at the BLAST e-value of 1e–5, compared to the direct BLAST against NCBI-nr database. The annotation results of SAP are exactly in agreement with those of the direct NCBI-nr database BLAST-MEGAN approach, which is very time-consuming and computationally intensive. Selecting rigorous thresholds (e.g. e-value of 1e–10) would further accelerate SAP process. The SAP pipeline may also be coupled with novel similarity search tools (e.g. RAPsearch) other than BLAST to achieve even faster annotation of huge metagenomic datasets. Above all, this sub-database construction method and SAP pipeline provides a new time-efficient and convenient annotation similarity search strategy for laboratories without access to high performance computing facilities. SAP also offers a solution to high performance computing facilities for the processing of more similarity search tasks.

## Introduction

High-throughput sequencing (HTS), such as 454 pyrosequencing and Illumina sequencing, have been recently applied as novel promising methods to investigate genes or gene expression of microbial communities in different habitats, such as marine water [1], soil [2], human guts [3], oral cavities [4], and activated sludge [5], [6]. HTS-metagenomic/metatranscriptomic approaches allow researchers to obtain more genetic information from environmental samples, but they also pose new challenges in data analysis. As one of these challenges, the demand on computational resources has become one of the bottlenecks for metagenomics projects [7]. For a typical analysis, the metagenomic dataset (e.g. reads or open reading frames) would be firstly subjected to similarity search against certain databases, followed by annotation of the output using some other tools, for example, MEGAN. BLAST is the most commonly used similarity search tool that is designed to find distant homologous sequences for taxonomic and functional attributes [8], but requires tremendous computational capacity. For instance, it will take a month for a 1000-CPU computer cluster to conduct a full BLASTX search against the whole NCBI-nr database (amino acid sequences of ∼4 Gigabytes (GB)) for a 20 Giga base pairs (Gbp) DNA dataset [8]. Based on our test, it took approximately 3 weeks to search a set of 100 Mbp DNA against NCBI-nr database using a BLASTX on a workstation (Lenovo ThinkStation-D20: CPU 2.40 GHz×16 threads; Memory 96 GB). It will be a great challenge for those laboratories without access to super-computers to analyze the huge HTS metagenomic dataset by BLASTX against NCBI-nr approach.

Various tools, e.g. PatternHunter [9], [10], BLAT [11], and BLASTZ [12], have been developed for fast similarity searching. However, these tools more or less sacrificed searching sensitivity comparing with BLAST. One recently developed tool, RAPsearch, attracted attention because it outperformed the BLASTX by ∼20–90 times in terms of speed; it missed only a small fraction (0.3∼3.2%) of BLASTX similarity hits and discovered additional homologous proteins (0.3∼2.1%) that BLASTX missed [8]. Besides the newly developed similarity searching tools, some online servers (e.g. IMG-M [13], CAMERA [14], and MG-RAST [15]) provide other solutions for individual research groups to handle huge metagenomic datasets. Equipped with taxonomic/functional assignment and pathway reconstruction, these online servers are serving as powerful tools in the fast annotation and visualization of metagenomic datasets both in specific details and as a whole [7]. However, these online platforms also suffer some drawbacks. For example, the IMG/M database consists of some microbial metagenome data integrated with isolate microbial genomes [13], but it is still limited when compared with the NCBI-nr database; MG-RAST version 3 relied on BLAT for similarity search, which is less sensitive than BLAST [9]. Owing to the dramatically increased amount of HTS metagenomic datasets submitted, these systems are also facing larger and larger computational burden and consequently cannot finish the analysis in a timely way. Such situation may get even worse in the future.

NCBI-nr, a non-redundant and comprehensive database, which is being updated frequently, contains both metabolic pathway information and functionally related taxonomic information. Searching against this database may provide more comprehensive annotation of the HTS metagenomic sequences, but it could be a waste of time and effort for those targeting specific pathways or functions, such as degradation pathways of some pollutants, nitrification/denitrification, since these sequences may account for a very small portion of the whole NCBI-nr database.

Using the specific sub-database for BLAST may largely reduce the computation time for the same size of DNA dataset, therefore lower the high demand on computational capacity in functional and taxonomic (based on functional gene) annotation.

However, the NCBI-nr database does not provide sub-databases with either specific function or metabolic pathways at this time being although it contains sub-databases of different taxonomic groups. In the present study, we proposed a novel approach to extract specifically customized sub-databases from NCBI-nr database.

MEGAN is a powerful annotation tool that can visualize BLAST search results according to different annotation systems (including KEGG, Subsystem and Distribution) by translating the Gene ID numbers from the NCBI-nr database into their cryptic combined taxonomic and functional annotation [16], becoming a much more popular application than other annotation platforms. To construct the sub-databases in a very fast and simple way, we employed MEGAN to execute the translation/annotation process with an artificial file in BLAST format containing all the sequences in NCBI-nr and their corresponding GI numbers. Using MEGAN, we may easily extract the sub-databases in a high quality way according to different function/pathways, with little manual effort. With the constructed sub-databases, we further developed a three-step sub-database annotation pipeline (SAP) applying a two-step local BLAST to ensure the fast and accurate similarity search of huge metagenomic datasets. The methods developed in the present study require much less computational capacity and are very time-efficient, thus are suitable for those laboratories without high performance computing facilities. The method may also help high performance computing facilities to execute more similarity search tasks.

## Results

### Sub-database Construction Using the NCBI-nr Database and MEGAN

**Figure 1:**
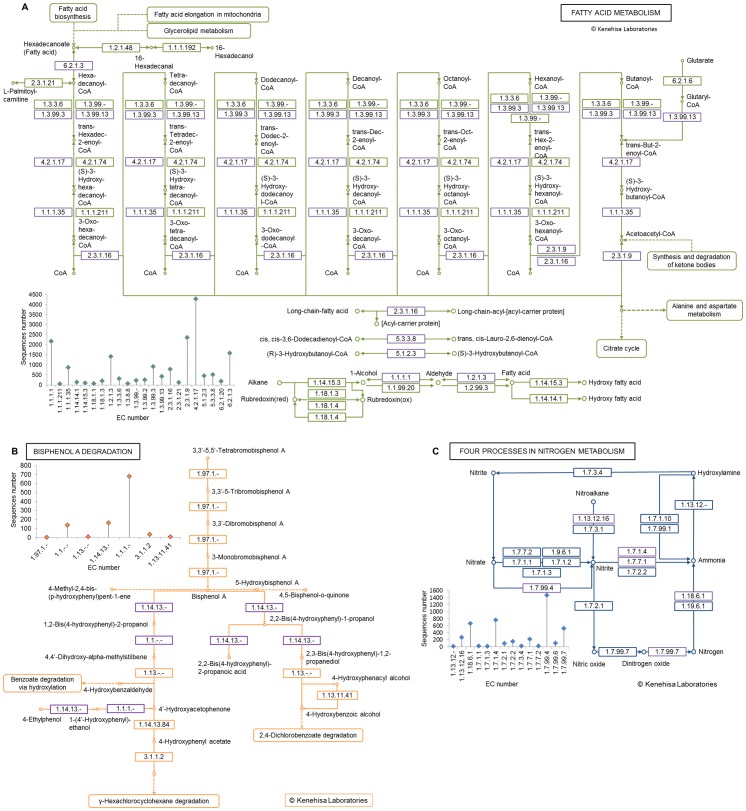
The maps of sub-databases constructed by the proposed method. 16089, 1023, and 4318 NCBI-nr database sequences were annotated to fatty acid metabolism pathway, bisphenol A degradation metabolism pathway, and the four processes in nitrogen metabolism, respectively. (A) Fatty acid metabolism pathway sub-database; (B) Sub-database of Bisphenol A degradation pathway; (C) The four processes in nitrogen metabolism. The bar in the figures showed the number of sequences annotated to the EC numbers. The EC numbers with relative high counts were highlighted in purple.

After importing the artificial tabular BLAST output into MEGAN, the MEGAN-KEGG mapper showed that 16089, 1023, and 4318 sequences from the NCBI-nr database were annotated to fatty acid metabolism pathway, bisphenol A degradation metabolism pathway, and the four processes in nitrogen metabolism, respectively (Figure 1). Table 1 shows the numbers of the NCBI-nr database sequences annotated to EC numbers in the four processes in nitrogen metabolism. The numbers of sequences annotated to EC numbers in the other two pathways are shown in Table S1.

**Table 1:** Number of sequences derived from NCBI-nr database, which were annotated to the ammonification, de-nitrification, nitrification, and nitrogen fixation processes in nitrogen metabolism by MEGAN.

Ammonification, de-nitrification, nitrification, and nitrogen fixation processes in nitrogen metabolism				
EC number	KO number	Name	Definition	Number of sequences derived from the NCBI-nr database
1.13.12.-	K10944	amoA	Ammonia monooxygenase subunit A	3
	K10945	amoB	Ammonia monooxygenase subunit B	2
	K10946	amoC	Ammonia monooxygenase subunit C	7
1.13.12.16	K00459	E1.13.12.16	Nitronate monooxygenase	267
1.18.6.1	K00531	anfG	Nitrogenase	13
	K02588	nifH	Nitrogenase iron protein NifH	247
	K02586	nifD	Nitrogenase molybdenum-iron protein alpha chain	195
	K02591	nifK	Nitrogenase molybdenum-iron protein beta chain	209
1.7.1.1	K00360	E1.7.1.1	Nitrate reductase (NADH)	21
1.7.1.3	K10534	NIAD	Nitrate reductase (NADPH)	6
1.7.1.4	K00362	nirB	Nitrite reductase (NAD(P)H) large subunit	457
	K00363	nirD	Nitrite reductase (NAD(P)H) small subunit	303
1.7.2.1	K00368	E1.7.2.1	Nitrite reductase (NO-forming)tabl	96
1.7.2.2	K03385	nrfA	Cytochrome c-552	147
1.7.3.4	K10535	hao	Hydroxylamine oxidase	16
1.7.7.1	K00366	nirA	Ferredoxin-nitrite reductase	216
1.7.7.2	K00367	narB	Ferredoxin-nitrate reductase	20
1.7.99.4	K00369	narX	Nitrate reductase-like protein	14
	K00370	narG	Nitrate reductase 1, alpha subunit	260
	K00371	narH	Nitrate reductase 1, beta subunit	239
	K00372	E1.7.99.4C	Nitrate reductase catalytic subunit	220
	K00373	narJ	Nitrate reductase 1, delta subunit	226
	K00374	narI	Nitrate reductase 1, gamma subunit	221
	K02567	napA	Periplasmic nitrate reductase NapA	183
	K08345	narZ	Nitrate reductase 2, alpha subunit	35
	K08346	narY	Nitrate reductase 2, beta subunit	28
	K08347	narV	Nitrate reductase 2, gamma subunit	24
1.7.99.6	K00376	nosZ	Nitrous-oxide reductase	98
1.7.99.7	K02164	norE	Nitric oxide reductase NorE protein	53
	K02305	norC	Nitric oxide reductase subunit C	67
	K02448	norD	Nitric oxide reductase NorD protein	67
	K04561	norB	Nitric oxide reductase subunit B	210
	K04747	norF	Nitric oxide reductase NorF protein	4
	K04748	norQ	Nitric oxide reductase NorQ protein	123

### Verification of SAP against the Direct BLAST Using the Whole NCBI-nr Database

SAP was verified by comparing the MEGAN annotation results of SAP-BLAST outputs with the MEGAN annotation results of direct NCBI-nr BLAST outputs. For both approaches, BLAST was first conducted using two e-value cutoffs (1e–5 and 1e–10) and MEGAN was then applied to annotate BLAST output with default parameters. An activated sludge metagenomic dataset was used in this test.

The extracted sub-database functioned as a filter to first catch possible homologous sequences. As shown in Table 2, annotated reads of SAP 1^st^ BLAST were much more than those of SAP 2^nd^ BLAST and NCBI-nr BLAST at different cutoffs. Using an e-value of 1e–5, which is commonly used in various literature [5], [17], [18], the SAP 1^st^ BLAST found 1∼2 fold of homogeneous reads comparing with those after the SAP 2^nd^ BLAST and the direct NCBI-nr BLAST for most enzymes. In some cases, e.g. EC 1.13.12.-, EC 1.13.12.16, and EC 1.14.15.3, SAP 1^st^ BLAST found even 2∼4 times more reads than the SAP 2^nd^ BLAST and the direct NCBI-nr BLAST.

**Table 2:** Comparison of the number of sequences annotated by SAP with direct BLAST against the NCBI-nr database.

EC number	SAP 1^st^ BLAST	SAP 2^nd^ BLAST	BLAST against nr	EC number	SAP 1^st^ BLAST	SAP 2^nd^ BLAST	BLAST against nr
Fatty acid metabolism pathway							
1.1.1.1	37	24	24	1.14.15.3	9	3	3
1.1.1.35	55	43	43	1.18.1.3	1	0	0
1.2.1.3	91	53	53	2.3.1.16	11	8	8
1.3.3.6	2	2	2	2.3.1.9	67	52	52
1.3.99.-	3	3	3	4.2.1.17	59	48	48
1.3.99.2	45	11	11	5.1.2.3	16	13	13
1.3.99.3	90	40	40	5.3.3.8	17	13	13
1.3.99.7	24	20	20	6.2.1.3	100	50	50
1.3.99.13	10	7	7	6.2.1.20	3	3	3
1.14.14.1	5	2	2				
Bisphenol A degradation pathway							
1.1.-.-	7	4	4	1.14.13.-	21	5	5
1.1.1.-	61	16	16				
Ammonification, de-nitrification, nitrification, and nitrogen fixation processes in nitrogen metabolism							
1.13.12.-	6	2	2	1.7.1.4	14	13	13
1.13.12.16	5	1	1	1.7.2.1	11	5	5
1.18.6.1	5	5	5	1.7.99.6	15	10	10
1.7.1.1	1	1	1	1.7.99.7	26	19	19

The SAP 2^nd^ BLAST annotation results were completely in agreement with the direct NCBI-nr BLAST annotation results of the three selected pathways for all the EC numbers. At an e-value of 1e–5, both the SAP 2^nd^ BLAST annotation results and the direct NCBI-nr BLAST annotation results contained 790 reads, 50 reads, and 139 reads annotated to fatty acid metabolism pathway, bisphenol A degradation pathway, and the four processes in nitrogen metabolism pathway, respectively (Table 2). Additionally, the SAP 2^nd^ BLAST annotation results were also exactly the same as the direct NCBI-nr BLAST annotation results under an e-value of 1e–10 in all the three sub-databases, showing great consistency between the SAP 2^nd^ BLAST annotation and the direct NCBI-nr BLAST annotation.

### Time Efficiency of SAP Annotation

The test results in the present study showed that SAP largely shortened the time cost of specific pathway annotation. Table 3 shows that 300,000 reads of the test activated sludge metagenomic dataset consumed 150±0.4 CPU hours on a workstation (described in Methods section) to obtain the BLAST output. In comparison, at an e-value of 1e–5, only 0.99±0.05, 0.39±0.02, and 0.58±0.04 CPU hours were consumed by the whole SAP (1^st^ and 2^nd^) BLAST against fatty acid metabolism, bisphenol A degradation, and the four processes in nitrogen metabolism sub-databases, respectively (Table 3). Depending on the sizes of different sub-databases, time consumption of SAP was ∼150–385 times lower than direct BLAST against the whole NCBI-nr database.

**Table 3:** Time consumption in SAP annotation and direct the NCBI-nr annotation.

Testdataset	Number of reads	Read length (nt)	Sub-database	BLAST e-value	Subjects number of sub-database	Running time (CPU hours)				Fold increase in speed
						SAP (1^st^ step)	SAP (2^nd^ step)	SAP total	NCBI-nr annotation	
SL_DNA	300,000	100	FA	1e–5	16,089	0.13±0.01	0.86±0.05	0.99±0.05	150±0.4	152
			N		4,318	0.16±0.01	0.42±0.03	0.58±0.04		259
			BPA		1,023	0.03±0	0.36±0.02	0.39±0.02		385
			FA	1e–10	16,089	0.13±0.01	0.22±0.01	0.35±0.02	150±0.4	429
			N		4,318	0.16±0.01	0.08±0	0.24±0.01		625
			BPA		1,023	0.01±0	0.04±0	0.05±0		3000

Fold increase in speed refers to the speedup achieved by SAP as compared to the direct NCBI-nr blast.

Further tests showed that the stricter e-value resulted in smaller 1^st^ BLAST output, thereby accelerating 2^nd^ BLAST. At an e-value of 1e–10, SAP was ∼429–3000 times faster than direct BLAST against the NCBI-nr database (Table 3).

## Discussion

### Construction of Sub-databases

Since a sub-database contains much less target sequences than the whole NCBI-nr database, significant speedup could be expected in a similarity search (BLAST) against a specific sub-database compared with BLAST against the NCBI-nr database containing largely irrelevant target sequences. However, the NCBI-nr database does not provide the sub-databases for different specific groups (pathway, subsystem, etc.) at this time being. The present study developed a method to simply extract specific sub-database from the NCBI-nr database via MEGAN for quick similarity searching firstly against the sub-database and secondly against the NCBI-nr database by two sequential BLAST operations before the final MEGAN annotation.

The proposed sub-database construction method was based on the understanding of the KEGG database, the NCBI-nr database and MEGAN. Both the KEGG database and the NCBI-nr database were frequently adopted for BLAST similarity searches. The NCBI-nr has been widely used as a database in similarity searching of metagenomic datasets for functional annotation [2] since it is a non-redundant, comprehensive and frequently updated database that contains both information on metabolic pathways and function-gene-based taxonomic annotation. The NCBI-nr BLAST output is usually further annotated by MEGAN. Besides taxonomic analysis and functional assignment, MEGAN supports automatically mapping the NCBI-nr BLAST output to the KEGG pathway nodes, and comparison of discrepancies among different datasets, which is convenient for metagenomic researchers. Based on this, the present study selected the NCBI-nr database instead of KEGG as the mother database from which to extract and construct sub-database. MEGAN was used as a filter to collect target sequences and generate a list of GI numbers according to a specific pathway. After that, the GI numbers were used to extract sub-database sequences from the NCBI-nr database.

The proposed sub-database construction method using MEGAN requires little manual effort and is quick. Sub-databases containing target sequences could be extracted from the NCBI-nr database within several minutes to several hours, depending on the sequence numbers within the sub-databases. This sub-database construction method is also easy to follow and could be performed flexibly. For instance, researchers who are interested in certain physiological/biochemical processes, e.g. denitrification process in nitrogen metabolism, can easily extract all the sequences related to the process (EC 1.13.12.- and EC 1.7.3.4) from the NCBI-nr database using the method proposed in the present study. The proposed sub-database construction method may help researchers construct the customized databases in a very short time, without any requirement for experience of database construction.

### SAP vs. Direct BLAST against the NCBI-nr Database

The three-step SAP based on the constructed sub-databases in the present study could quickly annotate the metagenomic dataset. SAP 2^nd^ BLAST was performed as an effective double-checking way to verify the results from 1^st^ BLAST of SAP. Coupled 1^st^ and 2^nd^ BLAST in SAP may give the exactly same annotation results as those of direct NCBI-nr BLAST under the same cutoff. Results under different cutoffs showed great consistency between SAP-MEGAN approach and NCBI-nr direct BLAST-MEGAN approach.

SAP enables faster similarity search compared to a direct search against the NCBI-nr database. The tests of SAP using the selected sub-databases and the dataset remarkably achieved a speedup of ∼152–385 times compared with direct NCBI-nr BLAST at an e-value of 1e–5. Previous studies reported novel similarity search tools (e.g. BLAT) enabled faster similarity search, and several commercial accelerated-BLAST software applications (e.g. MBLAST, MAPX, etc.) claimed the software could achieve a speed improvement of 100–1000× comparing with BLAST. Current tools development generally based on the modification or redesign of the similarity search algorithm, whereas investigators have over-looked the advantage of breaking up the huge database into small sub-databases. The present proposed method provides a new strategy that not only guarantees time efficiency and ensures convenient annotation, but also reduces similarity search cost. In addition, the SAP method could be flexibly coupled with any newly developed similarity search tool, e.g. RAPsearch, which can run ∼20–90 times faster than BLAST [8]. Combining such tools will make similarity searching with SAP even faster, providing a quicker route to investigating the pathways in huge metagenomic datasets.

The computation time required by the SAP 2^nd^ BLAST is about ∼4–18× of that by the SAP 1^st^ BLAST according to the tests conducted, suggesting that the 2^nd^ BLAST is the rate-limiting step in SAP. Time consumption of SAP 2^nd^ BLAST largely depends on the number of hit sequences in the output of SAP 1^st^ BLAST. SAP 1^st^ BLAST, as a filter to screen out sequences with low similarities to the target sequences, is crucial for reducing the time required for 2^nd^ BLAST. Using a rigorous threshold (e.g. similarity, alignment length, e-value) could sharply reduce the number of hit sequences in SAP 1^st^ BLAST, therefore decreasing the time consumption in SAP 2^nd^ BLAST. The test results showed that SAP achieved a speedup of ∼2.8–7.8 times against the three sub-databases under the e-value of 1e–10 than under the e-value of 1e–5 (∼429–3000 times faster than direct BLAST against the NCBI-nr database at the e-value of 1e–10). For a big set of metagenomic data, applying a stricter e-value would further significantly reduce the time consumption of SAP.

### Limitations of the Methodology Proposed in the Present Study

The proposed sub-database construction method and the SAP approach have their limitations, due to its reliance on MEGAN. For example, the sub-database construction method is unable to construct a sub-database for subject sequences that are not included in MEGAN KEGG mapper, MEGAN SEED subsystem categories and MEGAN Distribution categories. Also the functional and taxonomic information in MEGAN must be kept up to date. However, MEGAN is still one of the most popular tools for the fast and convenient annotation of the BLAST results of huge metagenomic datasets against the NCBI-nr database. It enables combination of taxonomic analysis with functional analysis (SEED and KEGG classification, and provides additional analyzed information (e.g. microbial distribution) of metagenomic datasets, making it a very powerful metagenomic analyzer. Nevertheless, MEGAN would become more complete and accurate with further improvements. As a matter of fact, the developer released their most update version of gi_taxid_nucl/prot files in July 2012, showing their continuous effort to make it more consistent with the KEGG and NCBI-nr databases.

Another possible way to utilize SAP is to BLAST the metagenomic datasets against a sub-database directly extracted from the KEGG/SEED database first, and then BLAST the extracted hit reads against the NCBI-nr database. Taking the KEGG database as an example, scripts were written to exclude sequences assigned to other pathway/biological process and summarize the final annotation results according to KO numbers that could be obtained from the KEGG pathway website. However, the final results must be manually added to a self-made KEGG map, and the pairing relationship between KO number and GI number is still needed. Alternatively, the BLAST output could be uploaded to the KEGG website for visualization of the pathway, but without taxonomic information of these functional genes and other combined annotation available in MEGAN. Although these two approaches could avoid the possible deficiency of sub-database constructed based on MEGAN, they require much manual effort and custom scripts for annotation or visualization.

In summary, the present study developed a fast method to construct local sub-databases from the NCBI-nr database for quick similarity search and annotation of huge metagenomic datasets based on BLAST-MEGAN approach. A three-step sub-database annotation pipeline was further proposed to conduct the annotation in a time-efficient and user-friendly way that requires much less computational capacity than the direct NCBI-nr database BLAST-MEGAN approach. The pipeline achieved a speedup of ∼150–385 times at an e-value of 1e–5 compared with direct BLAST against the NCBI-nr database,and the SAP 2^nd^ BLAST annotation results were exactly in agreement with the direct NCBI-nr BLAST annotation results. The selection of a rigorous e-value threshold (e.g. e-value: 1e–10) would accelerate sub-database annotation by SAP with a speedup of ∼429–3000 times when compared with direct BLAST against the NCBI-nr database, depending on the size of sub-database. SAP may also be coupled with other novel similarity search tools other than BLAST (e.g. RAPsearch) to achieve even faster annotation of huge metagenomic datasets. Although the proposed methods also have their limitations, the sub-database construction method and SAP are especially useful for those laboratories without high-performance computing facility to handle their metagenomic dataset, because the methods are not computationally intensive, easy to follow, and could be performed flexibly.

## Methodology

### Sub-database Construction Using MEGAN

**Figure 2:**
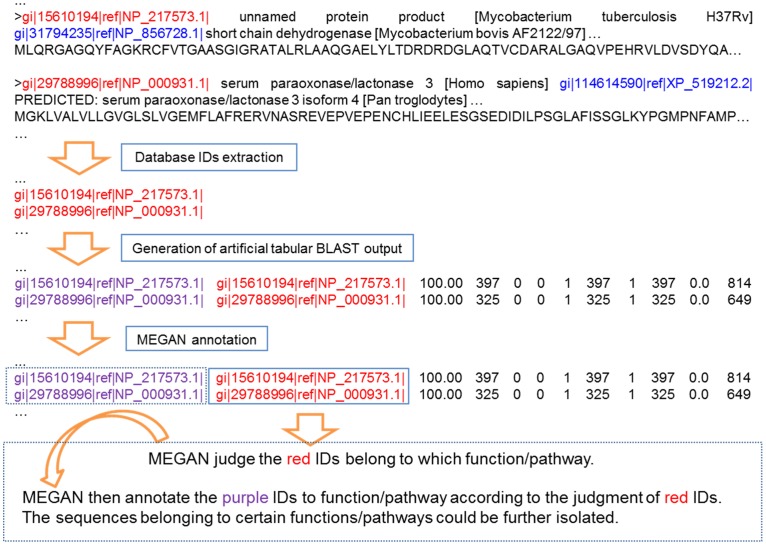
Procedure of sub-database construction using MEGAN.

Three factors are important for MEGAN to parse tabular BLAST output: query ID, subject ID, and the bit-score of each item. MEGAN annotates/assigns the query (with unique query ID) to different taxa, SEED subsystem categories or KEGG metabolic pathways according to the corresponding subject ID of an item. As shown in Figure 2, we extracted subject IDs (the first GI number of a subject) from the NCBI-nr database (in FASTA format), and then converted them into an artificial tabular BLAST output file, in which each item used the GI number as its query ID and subject ID, plus arbitrary setup of bit-scores higher than the default bit-score (e.g. >35) of MEGAN using a custom Python script, as shown in the following example: “*gi|22536352|ref|NP_687203.1* (query ID) *gi|22536352|ref|NP_687203.1* (subject ID) *100.00 397 0 0 1 397 1 397 0.0 814* (bitscore)”.

MEGAN annotated these artificial items and assigned all these subjects in the NCBI-nr database into different taxa, functional groups, and pathways. Then the GI numbers assigned to the target group (could be a taxon, a functional group, or a pathway) were extracted from MEGAN and the sub-databases with the sequences could be further extracted from the NCBI-nr database using the list of these GI numbers.

In the present study, three sub-databases, that is, fatty acid (FA) metabolism, bisphenol A (BPA) degradation, and four biochemical processes in nitrogen metabolism (N), were extracted from the NCBI-nr database (ftp://ftp.ncbi.nlm.nih.gov/blast/db/FASTA/). Fatty acid metabolism is a fundamental pathway that widely exists in cellular organisms. Bisphenol A degradation is a representative of xenobiotics biodegradation and metabolism, a common research area in environmental science and engineering filed. The third sub-database containing sequences associated with four processes (ammonification, de-nitrification, nitrification, and nitrogen fixation) in nitrogen metabolism, were selected due to their significance in global nitrogen cycle and nitrogen removal in biological wastewater treatment [5].

The scripts using in the present study could be download from Dr. Zhang Tong’s webpage (http://web.hku.hk/~zhangt/ZhangT.htm).

### Validation of BLAST Results of Sub-database

**Figure 3:**
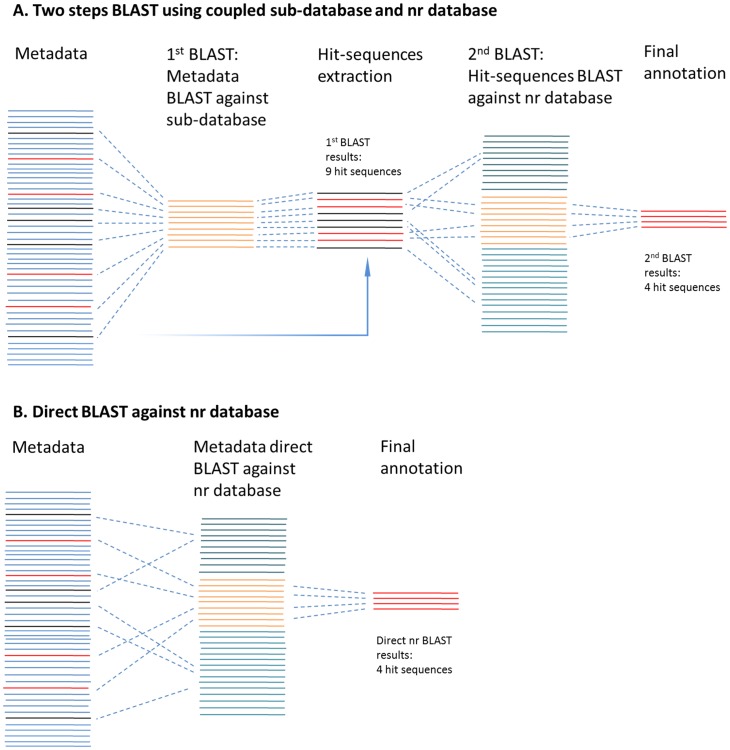
Sub-database annotation pipeline. A) SAP pipeline: two steps BLAST using coupled sub-database and the NCBI-nr database; B) Direct BLAST against the NCBI-nr database.

BLAST output using an extracted sub-database might be inaccurate for some query sequences because it may not contain their best similar subject sequences, but these subject sequences could be present in the larger NCBI-nr database, which contains much more reference sequences than the extracted sub-database. To solve this problem and validate the BLAST results of sub-database, a three-step sub-database annotation pipeline, i.e. two-step BLAST approach plus the final MEGAN annotation step (Figure 3), was proposed in the present study to provide fast similarity search and accurate annotation.

In detail, the dataset was firstly BLAST against the target sub-database, which functioned as a filter to quickly search all the possible hit query sequences from the dataset. In the next step, the hit query sequences were isolated from the dataset, and then used for the 2^nd^ BLAST against the whole NCBI-nr database. Then, the 2^nd^ BLAST results were annotated using MEGAN and the query sequences assigned to the target groups (taxon, function, pathway, etc.). If there were query sequences assigned to groups other than the target group, these sequences would be excluded in MEGAN annotation and discarded in the final summary to guarantee the accuracy of the similarity search results.

### Verification of SAP against the Direct BLAST Using the Whole NCBI-nr Database

The proposed pipeline, SAP, was verified by comparing the annotation results of SAP with direct BLAST against the whole NCBI-nr database. A dataset of 300,000 DNA reads (100 bp) was randomly isolated from a ∼2.4 Gbp activated sludge DNA dataset, which could be downloaded from MG-RAST server (MG-RAST ID: 4467420.3). The activated sludge sample used for DNA extraction was collected from the aeration tank of a local wastewater treatment plant (Stanley) in Hong Kong. No specific permits were required for the described field studies. We a.) confirm that the location is not privately-owned or protected in any way; b.) confirm that the field studies did not involve endangered or protected species.

The results using the SAP-MEGAN approach were compared with the results of the direct BLAST-MEGAN against the NCBI-nr database to verify the accuracy of the SAP approach. Time consumption of both the SAP and direct NCBI-nr BLAST were calculated and compared on a 16-core workstation (Lenovo ThinkStation-D20: CPU Intel® Xeon(R) E5620@ 2.40 GHz ×16; Memory 96 GB).

## Supporting Information

Table S1 **Number of sequences derived from NCBI-nr database, which were annotated to the fatty acid metabolism pathway and bisphenol A degradation metabolism pathway.**
(DOCX)
