# Supplementary material for: Construction of Customized Sub-Databases from NCBI-nr Database for Rapid Annotation of Huge Metagenomic Datasets Using a Combined BLAST and MEGAN Approach
Source: PLoS One. 2013 Apr 1;8(4):e59831. doi: 10.1371/journal.pone.0059831 (PMC3613424; doi:10.1371/journal.pone.0059831)
Supplement: Table S1 — Number of sequences derived from NCBI-nr database, which were annotated to the fatty acid metabolism pathway and bisphenol A degradation metabolism pathway. (DOCX) [file pone.0059831.s001.docx]

Table S1. Number of sequences derived from NCBI-nr database, which were annotated to the fatty acid metabolism pathway and bisphenol A degradation metabolism pathway.

| EC number | KO number | Name | Definition | Number of sequences derived from the NCBI-nr database |
| --- | --- | --- | --- | --- |
| **Fatty acid metabolism pathway sub-database** | | | | |
| 6.2.1.3 | K01897 | ACSL, fadD | long-chain acyl-CoA synthetase | 1583 |
| 2.3.1.21 | K08765 | CPT1 | carnitine O-palmitoyltransferase 1 | 68 |
|  | K08766 | CPT2 | carnitine O-palmitoyltransferase 2 | 51 |
| 1.3.3.6 | K00232 | E1.3.3.6 | acyl-CoA oxidase | 306 |
| 1.3.99.3 | K00249 | ACADM | acyl-CoA dehydrogenase | 907 |
| 1.3.99.13 | K00255 | ACADL | long-chain-acyl-CoA dehydrogenase | 68 |
| 1.3.99.- | K09479 | ACADVL | very long chain acyl-CoA dehydrogenase | 25 |
|  | K06445 | fadE | acyl-CoA dehydrogenase | 195 |
| 1.3.99.2 | K00248 | ACADS | butyryl-CoA dehydrogenase | 252 |
| 1.3.99.7 | K00252 | GCDH | glutaryl-CoA dehydrogenase | 416 |
| 4.2.1.17 | K01692 | paaF, echA | enoyl-CoA hydratase | 3687 |
|  | K01825 | fadB | 3-hydroxyacyl-CoA dehydrogenase / enoyl-CoA hydratase / 3-hydroxybutyryl-CoA epimerase / enoyl-CoA isomerase | 136 |
|  | K01782 | fadJ | 3-hydroxyacyl-CoA dehydrogenase / enoyl-CoA hydratase / 3-hydroxybutyryl-CoA epimerase | 316 |
|  | K07511 | ECHS1 | enoyl-CoA hydratase | 45 |
|  | K07514 | EHHADH | enoyl-CoA hydratase / 3-hydroxyacyl-CoA dehydrogenase / 3,2-trans-enoyl-CoA isomerase | 12 |
|  | KO7515 | HADHA | enoyl-CoA hydratase / long-chain 3-hydroxyacyl-CoA dehydrogenase | 50 |
|  | K13767 | ysiB, fadB | enoyl-CoA hydratase | 26 |
| 1.1.1.35 | K00022 | HADH | 3-hydroxyacyl-CoA dehydrogenase | 65 |
|  | K07516 | fadN | 3-hydroxyacyl-CoA dehydrogenase | 334 |
|  | K07514 | EHHADH | enoyl-CoA hydratase / 3-hydroxyacyl-CoA dehydrogenase / 3,2-trans-enoyl-CoA isomerase | 12 |
|  | K01825 | fadB | 3-hydroxyacyl-CoA dehydrogenase / enoyl-CoA hydratase / 3-hydroxybutyryl-CoA epimerase / enoyl-CoA isomerase | 136 |
|  | K01782 | fadJ | 3-hydroxyacyl-CoA dehydrogenase / enoyl-CoA hydratase / 3-hydroxybutyryl-CoA epimerase | 316 |
| 1.1.1.211 | K07515 | HADHA | enoyl-CoA hydratase / long-chain 3-hydroxyacyl-CoA dehydrogenase | 50 |
| 2.3.1.16 | K00632 | E2.3.1.16 | acetyl-CoA acyltransferase | 652 |
|  | K07508 | ACAA2 | acetyl-CoA acyltransferase 2 | 47 |
|  | K07509 | HADHB | acetyl-CoA acyltransferase | 44 |
|  | K07513 | ACAA1 | acetyl-CoA acyltransferase 1 | 37 |
| 2.3.1.9 | K00626 | E2.3.1.9, atoB | acetyl-CoA C-acetyltransferase | 2361 |
| 6.2.1.20 | K01909 | mbtM | long-chain-fatty-acid--[acyl-carrier-protein] ligase | 18 |
|  | K05939 | Aas | acyl-[acyl-carrier-protein]-phospholipid O-acyltransferase / long-chain-fatty-acid--[acyl-carrier-protein] ligase | 165 |
| 5.3.3.8 | K01825 | fadB | 3-hydroxyacyl-CoA dehydrogenase / enoyl-CoA hydratase / 3-hydroxybutyryl-CoA epimerase / enoyl-CoA isomerase | 136 |
|  | K13238 | DCI | 3,2-trans-enoyl-CoA isomerase, mitochondrial | 51 |
|  | K13239 | PECI | peroxisomal 3,2-trans-enoyl-CoA isomerase | 42 |
|  | K07514 | EHHADH | enoyl-CoA hydratase / 3-hydroxyacyl-CoA dehydrogenase / 3,2-trans-enoyl-CoA isomerase | 12 |
|  | K01782 | fadJ | 3-hydroxyacyl-CoA dehydrogenase / enoyl-CoA hydratase / 3-hydroxybutyryl-CoA epimerase | 316 |
| 5.1.2.3 | K01782 | fadJ | 3-hydroxyacyl-CoA dehydrogenase / enoyl-CoA hydratase / 3-hydroxybutyryl-CoA epimerase | 316 |
|  | K01825 | fadB | 3-hydroxyacyl-CoA dehydrogenase / enoyl-CoA hydratase / 3-hydroxybutyryl-CoA epimerase / enoyl-CoA isomerase | 136 |
| 1.1.1.1 | K13951 | ADH1_7 | alcohol dehydrogenase 1/7 | 31 |
|  | K13980 | ADH4 | alcohol dehydrogenase 4 | 6 |
|  | K13952 | ADH6 | alcohol dehydrogenase 6 | 4 |
|  | K13953 | adhP | alcohol dehydrogenase, propanol-preferring | 595 |
|  | K13954 | yiaY | alcohol dehydrogenase | 150 |
|  | K00001 | E1.1.1.1 | alcohol dehydrogenase | 560 |
|  | K00121 | frmA, ADH5, adhC | S-(hydroxymethyl)glutathione dehydrogenase / alcohol dehydrogenase | 567 |
|  | K04072 | adhE | acetaldehyde dehydrogenase / alcohol dehydrogenase | 259 |
| 1.2.1.3 | K00128 | E1.2.1.3 | aldehyde dehydrogenase (NAD+) | 1328 |
|  | K14085 | ALDH7A1 | aldehyde dehydrogenase family 7 member A1 | 58 |
|  | K00149 | ALDH9A1 | aldehyde dehydrogenase family 9 member A1 | 21 |
| 1.14.15.3 | K00496 | E1.14.15.3 | alkane 1-monooxygenase | 98 |
| 1.18.1.3 | K00529 | hcaD | ferredoxin--NAD+ reductase | 196 |
| 1.18.1.1 | K05297 | E1.18.1.1 | rubredoxin-NAD+ reductase | 62 |
| 1.14.14.1 | K00493 | E1.14.14.1 | unspecific monooxygenase | 130 |
| **Bisphenol A degradation pathway sub-database** | | | | |
| 1.97.1.- | K00539 | E1.97.1.- | -- | 2 |
| 1.1.-.- | K00120 | -- | -- | 135 |
| 1.14.13.- | K00492 | E1.14.13.- | -- | 161 |
| 1.1.1.- | K00100 | E1.1.1.- | -- | 678 |
| 3.1.1.2 | K01045 | PON | arylesterase / paraoxonase | 33 |
| 1.13.-.- | K05915 | E1.13.-.- | -- | 6 |
| 1.13.11.41 | K05913 | E1.13.11.41 | 2,4'-dihydroxyacetophenone dioxygenase | 8 |
